# Comparative genome‐scale analysis of *Pichia pastoris* variants informs selection of an optimal base strain

**DOI:** 10.1002/bit.27209

**Published:** 2019-11-12

**Authors:** Joseph R. Brady, Charles A. Whittaker, Melody C. Tan, D. Lee Kristensen, Duanduan Ma, Neil C. Dalvie, Kerry Routenberg Love, J. Christopher Love

**Affiliations:** ^1^ Koch Institute for Integrative Cancer Research Massachusetts Institute of Technology Cambridge Massachusetts; ^2^ Department of Chemical Engineering Massachusetts Institute of Technology Cambridge Massachusetts

**Keywords:** RNA‐Seq, yeast, recombinant protein, heterologous gene expression

## Abstract

*Komagataella phaffii*, also known as *Pichia pastoris*, is a common host for the production of biologics and enzymes, due to fast growth, high productivity, and advancements in host engineering. Several *K. phaffii* variants are commonly used as interchangeable base strains, which confounds efforts to improve this host. In this study, genomic and transcriptomic analyses of Y‐11430 (CBS7435), GS115, X‐33, and eight other variants enabled a comparative assessment of the relative fitness of these hosts for recombinant protein expression. Cell wall integrity explained the majority of the variation among strains, impacting transformation efficiency, growth, methanol metabolism, and secretion of heterologous proteins. Y‐11430 exhibited the highest activity of genes involved in methanol utilization, up to two‐fold higher transcription of heterologous genes, and robust growth. With a more permeable cell wall, X‐33 displayed a six‐fold higher transformation efficiency and up to 1.2‐fold higher titers than Y‐11430. X‐33 also shared nearly all mutations, and a defective variant of *HIS4*, with GS115, precluding robust growth. Transferring two beneficial mutations identified in X‐33 into Y‐11430 resulted in an optimized base strain that provided up to four‐fold higher transformation efficiency and three‐fold higher protein titers, while retaining robust growth. The approach employed here to assess unique banked variants in a species and then transfer key beneficial variants into a base strain should also facilitate rational assessment of a broad set of other recombinant hosts.

## Graphical abstract

*Pichia pastoris* is a common host for the production of biologics and enzymes, due to fast growth, high productivity, and advancements in host engineering. The authors assessed several *P. pastoris* variants for relative fitness as recombinant hosts by performing genomic, transcriptomic, and phenotypic analyses on each variant. This genome‐scale approach revealed key beneficial features, which were combined to create a novel, optimized base strain.

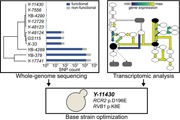

## 1 INTRODUCTION

Pipelines for recombinant biopharmaceuticals now include a growing number of forms and structures, including single‐domain antibodies, fusion proteins, antibody‐drug conjugates, bi‐specific antibodies, and subunit vaccines. This increasing diversity, coupled with increasing global demand for such products at reduced costs, present certain challenges for the host organisms commonly used today for production (Legastelois et al., 2017; Matthews et al., 2017). Rapidly advancing technologies for genomic engineering of hosts are promoting renewed consideration of microbial hosts for these tasks (Wagner & Alper, 2016). Selection of an optimal host for pursuing such purposes, however, can be difficult: many organisms may be suitable hosts, and for each organism, several variants typically exist (Jiang et al., 2019; Matthews et al., 2017). A framework for the rational evaluation of potential hosts could further promote the adoption of alternative hosts for commercial protein expression.

The methylotrophic yeast *Komagataella phaffii* (commonly known as *Pichia pastoris*) is one widely used candidate for the production of recombinant proteins. Detailed analyses of available strains could identify the genomic features that would inform an optimal base strain for deliberate engineering and development. *K. phaffii* is employed in the production of biosimilars, commercial innovator products, and several other molecules in development (Berlec & Štrukelj, 2013; EMA, 2018; Ghosh, 2017; Kuhlmann & Schmidt, 2014; Meehl & Stadheim, 2014). The yeast has been engineered to produce human‐like glycoforms (Hamilton et al., 2006). It has also achieved high volumetric productivity (Matthews et al., 2017), and offers potential for systematic genomic engineering to enhance productivity (Love, Dalvie, & Love, 2018). To date, *K. phaffii* has been used as a host in nearly 7,000 research articles since 2003 (Web of Science).

Despite the advantages of this host, the interchangeable use of various base strains of *P. pastoris* available today has led to reports of substantially variable performance. Productivity (Goncalves, 2013; Hochstrasser, Lüscher, De Virgilio, Boller, & Wiemken, 1998; Seo, & Rhee, 2004), growth (Hochstrasser et al., 1998; Seo, & Rhee, 2004; Sirén et al., 2006), proteolytic activity (Salamin, Sriranganadane, Léchenne, Jousson, & Monod, 2010; Sinha, Plantz, Inan, & Meagher, 2005; Sirén et al., 2006), and glycosylation (Blanchard et al., 2008; Tzeng & Jiang, 2004) have varied substantially among strains depending on the product expressed. Such inconsistencies underscore the need for a systematic evaluation of host properties to inform the choice of a standard base strain. Standardization should also enable streamlined host engineering for enhanced productivity and potentially facilitate greater adoption of *P. pastoris* as an alternative host for protein expression.

Here, we present a comprehensive assessment of the genomes, transcriptomes, and productivity of nine strains of *K. phaffii* available from the United States Department of Agriculture Culture Collection (USDA‐NRRL), as well as commercial strains GS115 and X‐33. Our analyses revealed significant variation among strains in genes related to cell wall integrity, and these genetic variations influence methanol metabolism, transformation efficiency, growth, and heterologous protein production. These differences may account, in part, for inconsistencies in the performance reported for this host. Y‐11430 performed best as a recombinant host, with high volumetric productivity and robust growth. X‐33 and GS115, however, offer advantages over Y‐11430 related to transformation efficiency (X‐33) and secretion of proteins (X‐33 and GS115), albeit with growth deficiencies. We found certain genomic features that account for aspects of these varied performances among strains, and provide a data‐driven approach for rational evaluation of alternative hosts and creation of a modified base strain of Y‐11430 for further development.

## 2 MATERIALS AND METHODS

### 2.1 Strains and cultivations

Y‐11430, Y‐48124, Y‐12729, Y‐48123, Y‐17741, Y‐7556, YB‐4290, YB‐378, and YB‐4289 were obtained from the USDA‐NRRL. X‐33 was purchased from Thermo Fisher Scientific and GS115 from ATCC 20864. Linear vectors containing genes with single‐nucleotide polymorphisms (SNPs) were integrated via homologous recombination to replace the native genes (*HIS4, RCR2*, or *RVB1*). Vectors contained the following (5′ to 3′): the open reading frame (ORF) with the SNP (5′ homology arm), the *AOX1* transcription terminator, a cassette for Blasticidin or G418 selection (Thermo Fisher Scientific), a bacterial replication origin, and the native terminator (3′ homology arm).

Strains were generated to express either human growth hormone (rhGH) or granulocyte‐colony stimulating factor (rhG‐CSF) under control of the *AOX1* promoter using a commercial vector (pPICZ A; Thermo Fisher Scientific, for gene sequences; see Table S1). Strains were generated to express trastuzumab with the heavy chain under control of the *AOX1* promoter and the light chain under control of the *DAS2* promoter. Competent cells were prepared and transformed as described elsewhere (Lin‐Cereghino et al., 2005). Cells were allowed to recover for 3 hr at 30°C without shaking and plated on YPD agar plates supplemented with 400 µg/ml phleomycin D1 (Thermo Fisher Scientific). For direct comparison of transformation efficiencies, strains independently were transformed with each of three vectors (either rhGH, rhG‐CSF, or rhIFN‐α2b) in duplicate for each input amount of DNA tested. Strains were grown in 24‐well deep well plates (25°C, 600 rpm) using glycerol‐containing media (BMGY‐Buffered Glycerol Complex Medium; Teknova) supplemented to 4% (v/v) glycerol. After 24 hr of biomass accumulation, cells were pelleted and resuspended in either fresh BMGY or BMMY (Buffered Methanol Complex Medium; Teknova) containing 1.5% (v/v) methanol. Samples from nontransgenic strains were collected after 24 hr initial growth in BMGY and after an additional 24 hr growth in either BMGY or BMMY. Samples from transgenic strains were collected after the additional 24 hr growth in BMMY. For screening growth without histidine, strains were grown using either synthetic complete (SC) or synthetic complete medium without histidine (SC‐his), prepared as described elsewhere (Sherman, 1991). Samples were collected every 12 hr from each of six independent replicates, with a final collection after 54 hr of growth.

### 2.2 Genome sequencing

The nine USDA‐NRRL strains and X‐33 were grown overnight in YPD (BD Difco). DNA was extracted as previously described (Lõoke, Kristjuhan, & Kristjuhan, 2011) and purified using the MagJET gDNA Kit (Thermo Fisher Scientific). Fragment libraries were prepared from genomic DNA as described previously (Love et al., 2016) and sequenced on an Illumina NextSeq to generate 150‐nt paired‐end reads.

Single‐nucleotide variant analysis was done according to the GATK Best Practices workflow (Van der Auwera et al., 2013). Alignments were performed using Burrows–Wheeler Aligner (BWA‐MEM) v0.7.5a (H. Li & Durbin, 2010), sorted, duplicates were marked, and .bam files were indexed using Picard v1.94 and SAMtools v0.1.19. Local realignment of high‐quality indels and SNPs was performed using GATK tools v3.1.1 and variants were functionally annotated with SnpEff v2.0.5d (Cingolani et al., 2012). A subset of five SNPs was confirmed by Sanger sequencing (Table S2). Insertions, deletions, and substitutions were identified using breseq v0.33.1 (Barrick et al., 2014) with bowtie2 v2.2.6 and R v3.3.1. Predicted translocations were identified using the function BND from DELLY v0.7.9 (Rausch et al., 2012) with the BWA‐MEM alignments as input.

### 2.3 Transcriptome analysis

RNA was extracted and purified according to the Qiagen RNeasy kit (cat. no. 74104) and RNA quality was analyzed to ensure RNA Quality Number >7. Nontransgenic strain RNA libraries were prepared using the Roche KAPA HyperPrep kit and sequenced on an Illumina NextSeq to generate 40‐nt paired‐end reads. RNA libraries for transgenic strains were prepared using the 3′ digital gene expression (3′ DGE) method (Soumillon, Cacchiarelli, Semrau, van Oudenaarden, & Mikkelsen, 2014) and sequenced on an Illumina HiSeq 2500 to generate paired reads of 17 bp (read 1) + 46 bp (read 2).

Sequenced single‐end reads from nontransgenic strains (KAPA method) were aligned and quantified using STAR v2.5.3a (Dobin et al., 2013) and RSEM v1.3.0 (B. Li & Dewey, 2011). Sequenced messenger RNA transcripts from transgenic strains (3′ DGE method) were quantified with Salmon v0.9.1 (Patro, Duggal, Love, Irizarry, & Kingsford, 2017), using a transcript database consisting of a single *K. phaffii* transcript per gene, each with a 100‐nt extension on the 3′ end, as well as rhGH and rhG‐CSF transgenes. Expression for both datasets was visualized using log_2_(transcripts per million [TPM] + 1) values. Sequencing data have been deposited in NCBI's Gene Expression Omnibus (GEO) and are accessible through GEO Series accession number GSE135666 (https://www.ncbi.nlm.nih.gov/geo/query/acc.cgi?acc=GSE135666).

Differential gene expression was analyzed using the DESeq2 package in R starting from gene integer counts and including log‐fold‐change shrinkage. Principal component analysis (PCA) was performed in R using prcomp with log_2_(TPM) values as input. The package PCGSE was used to analyze PCA loadings for enrichment of gene sets as described previously (Frost, Li, & Moore, 2015). Gene set enrichment analysis (GSEA) was performed in R using fgsea (Sergushichev, 2016) and GOseq (Young, Wakefield, Smyth, & Oshlack, 2010). GOseq analysis was performed with Bonferroni correction and Wallenius enrichment. ssGSEA was performed as previously described (Barbie et al., 2009) using GenePattern 2.0 (Reich et al., 2006). The reporter metabolites analysis was performed using RAVEN toolbox v1.08 and a published genome‐scale metabolic model for *K. phaffii* (Tomàs‐Gamisans, Ferrer, & Albiol, 2016). Additional R packages—UpSet (Lex et al., 2014), viridis, and plasma were used.

### 2.4 Analytical assays for strain characterization

Select strains were grown overnight in YPD and cell wall susceptibility assays were performed as described elsewhere (Ram & Klis, 2006). Congo red and Calcofluor white dyes (cat. nos. C6277 and F3543; Millipore Sigma) were added to final concentrations of 150 and 20 µg/mL, respectively.

Quantitative polymerase chain reaction (PCR) was performed according to PowerUp SYBR Green kit instructions (Thermo Fisher Scientific) using a Roche LightCycler 480. β‐Actin, rhGH, rhG‐CSF, and trastuzumab ORFs were PCR amplified and serially diluted to generate a standard curve (see Table S2 for primers). Experimental design and absolute copy number quantification were performed according to best practices (Abad et al., 2010)

Protein concentrations of rhGH and rhG‐CSF were determined using sandwich enzyme‐linked immunosorbent assay (ELISA) as described elsewhere (Crowell et al., 2018). rhGH‐specific antibodies were used at concentrations of 5 µg/ml capture (ab1954; Abcam, Cambridge, UK) and 0.6 µg/ml secondary/detection (ab1956; Abcam). rhG‐CSF‐specific antibodies were used at concentrations of 2 µg/ml capture (BVD13‐3A5; Biolegend, San Diego, CA), 0.4 µg/ml secondary (BVD11–37G10), and 0.2 µg/ml detection (ab7403; Abcam). Protein concentrations of trastuzumab were determined using a Human IgG1 ELISA kit (cat. no. RAB0242; Millipore Sigma).

## 3 RESULTS AND DISCUSSION

After nearly 30 years of use, the name *P. pastoris* was reclassified to include two distinct organisms: *Komagataella pastoris* (NRRL Y‐1603/CBS704) (Mattanovich et al., 2009) and *K. phaffii* (NRRL Y‐11430/CBS7435; (Küberl et al., 2011; Kurtzman, 2005, 2009; Sturmberger et al., 2016; Wegner, 1983). Early in its use, *K. phaffii* Y‐11430 was mutagenized with nitrosoguanidine to generate the histidine auxotroph, GS115, which became popular for the ease of integrating a heterologous gene into the genome using complementation of *HIS4* (Cregg, 1989; Cregg, Barringer, Hessler, & Madden, 1985; De Schutter et al., 2009). Later, X‐33 was reportedly created by complementation of *HIS4* into GS115 to restore prototrophy (Higgins et al., 1998), and is commercially available along with GS115. There are also several other strains of *K. phaffii* available in culture collections (https://nrrl.ncaur.usda.gov/) that may have utility as recombinant hosts. In total, there are at least 11 different historical strains excluding more recently engineered variants. To assess the relative fitness of each strain as a recombinant host, we first characterized the genomic and transcriptomic profiles of these 11 *K. phaffii* strains (Table 1).

**Table 1:** Identifying and source information for each strain characterized

Strain ID	Alternate ID	Source	Isolation information
Y‐11430	CBS 7435	USDA‐NRRL	Black oak, California
Y‐7556	CBS 2612	USDA‐NRRL	Black oak, California
YB‐4290	CBS 2612	USDA‐NRRL	Black oak, California
Y‐12729		USDA‐NRRL	Unknown, Mexico
Y‐48123		USDA‐NRRL	Unknown
GS115		Life Technologies^TM^	MNG mutant of Y‐11430
X‐33		Life Technologies^TM^	Revertant of GS115
Y‐48124	X‐33	USDA‐NRRL	Revertant of GS115
YB‐4289		USDA‐NRRL	Black oak, California
YB‐378		USDA‐NRRL	Elm
Y‐17741		USDA‐NRRL	Emory oak, Arizona

Abbreviations: MNG, methylnitronitrosoguanidine; USDA‐NRRL, United States Department of Agricultural‐Northern Regional Research Laboratory

### 3.1 Significant genetic variability exists among variants of *K. phaffii*

**Figure 1:**
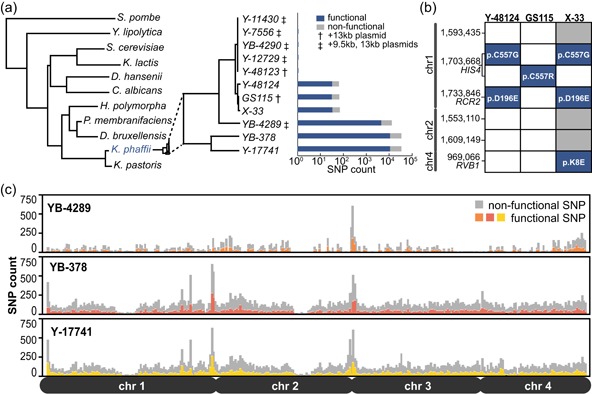
Genotypic comparison among wild‐type and biotechnological *Komagataella phaffii* strains. (a) Phylogeny and single‐nucleotide polymorphisms (SNP) count of *K. phaffii* strains sequenced in this study. SNP counts are shown on a logarithmic scale. The presence of either killer plasmid present in Y‐11430 is noted where applicable. (b) SNPs that differ between one or more members of the GS115 family (Group 2) strains. Gray and blue fill indicate the presence of nonfunctional and functional SNPs, respectively. The resultant missense is noted where applicable. (c) Distribution of SNPs across chromosomal positions for the three most variant strains (>16,000 SNPs, Group 3) [Color figure can be viewed at wileyonlinelibrary.com]

We hypothesized that conserved genetic polymorphisms among strains may cause phenotypic differences, so we sequenced the genome of each strain and performed variant calling based on alignment to our reference genome for Y‐11430 (Love et al., 2016). We analyzed the genomic sequences for the presence of functional and nonfunctional SNPs (Figure 1) and larger structural variants such as insertions or deletions relative to Y‐11430. We define functional SNPs to be polymorphisms that create a theoretical nonsynonymous mutation or impact gene expression or splicing. Genotyping revealed three dominant groups among these strains: ones with two or fewer variants relative to Y‐11430 (Group 1), GS115 and derivative strains (Group 2), and strains very different from Y‐11430 (Group 3; Figure 1a). Interestingly, several strains do not possess one or both of the linear plasmids present in Y‐11430 (Figure 1a), which may relate to the lineage of these strains.

Among the strains nearly identical to Y‐11430, we identified just a single SNP in four strains (Y‐12729, Y‐48123, Y‐7556, YB‐4290) relative to Y‐11430 (Figure 1a). Y‐7556 and YB‐4290 were identical genomically and contain a structural variant of the transcription factor (TF) Rsf2p with 183 additional C‐terminal amino acids compared to the Y‐11430 homolog. Interestingly, the Rsf2p homolog in *Saccharomyces cerevisiae* also possesses this C‐terminal extension, suggesting the stop codon in Y‐11430 was introduced after speciation (Lu, Roberts, Oszust, & Hudson, 2005). Given that Rsf2p is a TF implicated in cellular morphogenesis and alcohol metabolism, it is unsurprising that the methylotrophic *K. phaffii* Y‐11430 and non‐methylotrophic *S. cerevisiae* possess such a structural difference in this protein. In addition to the Rsf2p variant in Y‐7556/YB‐4290, we also identified a p.H2Y mutation in *SMC3* (chromatid cohesion) in Y‐12729, and a p.S315C mutation in *SEF1* (iron uptake) in Y‐48123.

We previously determined that GS115 possesses 74 SNPs relative to Y‐11430, 35 of which are potentially functional (Love et al., 2016). Several of the functional SNPs occur in DNA repair (*RAD5, EXO1*, and *DNL4*) or cell wall genes (*CWH43* and *GQ67_02041*). All 74 SNPs present in GS115 were also present in Y‐48124 (an NRRL banked strain of X‐33) and X‐33, with the exception of an amino acid variant at the highly conserved position 557 of the essential His4p protein (Love et al., 2016). Both Y‐48124 and X‐33 possess a p.C557G substitution in *HIS4*, rather than the p.C557R substitution found in GS115 (Figure 1b). This observed substitution contradicts the widespread reports in commercial kits (cat. no. C18000; Invitrogen) and the broader literature that X‐33 has a wild‐type *HIS4* genotype (Ahmad, Hirz, Pichler, & Schwab, 2014; Blanchard et al., 2008; Huang et al., 2014).

In addition to the 74 shared SNPs, variant calling also identified one functional SNP in Y‐48124, and five additional SNPs in X‐33 (Figure 1b). The additional mutation in Y‐48124, shared by X‐33, creates a p.D196E missense mutation in the endosomal vacuole protein Rcr2p, which sorts plasma membrane‐bound proteins (Kota, Melin‐Larsson, Ljungdahl, & Forsberg, 2007). Though not present in the conserved RCR domain, this mutation is located in a conserved region demonstrated to alter the stability of membrane proteins generally (Kota et al., 2007; Letunic & Bork, 2018). X‐33 also possesses a p.K8E missense mutation in the helicase protein, Rvb1p, which is responsible for a broad set of functions related to cell maintenance, including transcription, DNA repair, and the cell cycle (Jha & Dutta, 2009; Zhou et al., 2017). Taken collectively, these data corroborate a lineage from GS115 to Y‐48124 to X‐33, but do not support the hypothesis that X‐33 was created by simple complementation of *HIS4* in GS115 (Ahmad et al., 2014).

Three strains varied significantly from Y‐11430 (Figure 1a). We detected 16,000 SNPs in YB‐4289 and over 44,000 SNPs in YB‐378 and Y‐17741 relative to Y‐11430. Approximately one‐third of these SNPs are potentially functional and many are concentrated at the ends of chromosomes or in repetitive sequence regions, consistent with strain or organismal divergence (Figure 1c; Louis, Naumova, Lee, Naumov, & Haber, 1994). Despite these high SNP counts, these strains likely belong to *K. phaffii*, and not *K. pastoris*, since the latter species has on the order of one million SNPs relative to *K. phaffii* Y‐11430 (Love et al., 2016). Of note, YB‐378, YB‐4289, and Y‐17741 all possess the larger variant of Rsf2p present in Y‐7556 and *S. cerevisiae*.

In addition to SNPs, we detected several thousand structural variants (insertions, deletions, and substitutions) relative to Y‐11430 in these strains. The vast majority of these variants were less than 5 bp in length, but our analysis revealed larger variants such as a 4,000‐bp deletion in Y‐17741, two 1,000‐bp deletions in YB‐378 and Y‐17741, and a 276‐bp substitution in YB‐4289. We also found evidence of structural differences in several cell wall genes in these three strains relative to the other strains. Intragenic tandem repeats have been reported in cell wall genes in *S. cerevisiae* as a means to easily recombine and adapt to new environments (Verstrepen, Jansen, Lewitter, & Fink, 2005). Irregular depths of aligned reads in several flocculation genes (*ALS2, DIG, FLO1, FLO11‐MUC1, FLO11‐BSC1*, and two *FLO9*‐like genes) suggest a similar phenomenon in YB‐378, YB‐4289, and Y‐17741. Interestingly, X‐33 and Y‐48124 also display a similar structural difference in *ALS2* and *FLO11‐MUC1*, which may lead to a difference in the structure of the cell wall.

### 3.2 Transcriptomic characterization of strains reveals phenotypic clusters

**Figure 2:**
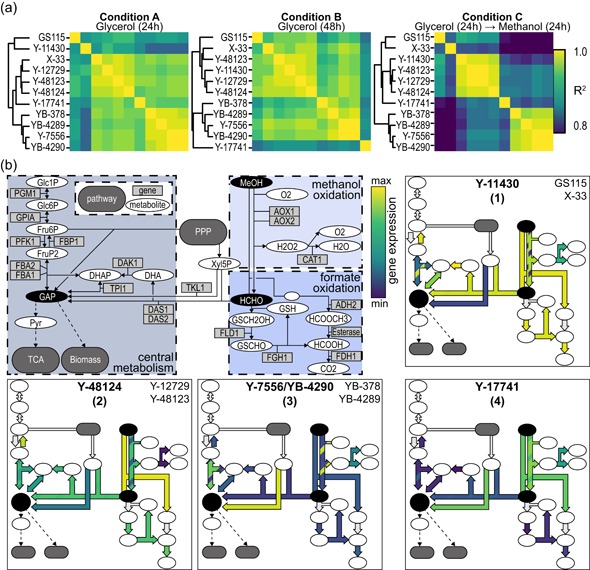
Whole transcriptome comparison of strains during different conditions of fermentation and expression of methanol utilization (Mut) pathway genes. (a) Correlation matrices for all expressed genes under each condition, with hierarchical clustering by Ward's method. (b) Depiction of the Mut pathway in *K. phaffii*, adapted from Küberl et al. (2011). Major intermediates are colored in black. Expression of Mut genes is illustrated for four strains representative of four different observed phenotypes; arrows for each pathway step are colored by the expression level of the appropriate enzyme(s) as determined by RNA‐Seq. Color scale indicates the relative expression of each gene across strains. When two genes contribute to a pathway step, the lower‐numbered gene is colored as the major stripe (e.g., *AOX1* is light green in Y‐11430) and the higher‐numbered gene is colored as the minor stripe (e.g., *AOX2* dark blue in Y‐11430) [Color figure can be viewed at wileyonlinelibrary.com]

With an understanding of the intrinsic genomic diversity of the strains, we next performed transcriptomic analysis on each strain to assess how the three groups varied in gene expression. We cultivated each strain in both glycerol‐containing and methanol‐containing media, which are commonly used to build biomass and express heterologous proteins, respectively (Life Technologies, 2014). We performed RNA‐Seq and measured cell growth (Figure S1) from samples collected under three relevant conditions: after growth for an initial 24 hr with glycerol (Condition A), after growth for an additional 24 hr with glycerol (Condition B), and after growth for an additional 24 hr with methanol (Condition C). We observed the expression of all genes under each of the three conditions, from which we determined the correlation of expression among strains (Figure 2a). In particular, gene expression was strongly correlated within each of two major clusters comprising Y‐11430, Y‐48124, Y‐12729, and Y‐48123 (Cluster 1) and Y‐7556/YB‐4290, YB‐378, and YB‐4289 (Cluster 2). The strong correlation within Cluster 2 was surprising given the thousands of SNPs present in YB‐378 and YB‐4289 relative to Y‐7556/YB‐4290. The correlation of expression between these two clusters was weak under Condition C, suggesting differences in growth with methanol. Further, we observed significant enrichment of gene sets related to central cell functions, such as RNA processing and translation, between these clusters under both Conditions A and C (Table S3). Gene expression in Y‐17741, the most divergent strain, was distinct from all other strains across all conditions. Differential expression analysis revealed nearly 1,000 differentially expressed genes (DEGs) between Y‐17741 and Cluster 1 under Conditions B and C (Figure S2). We leveraged these transcriptomic data, in conjunction with targeted assays, to evaluate each strain for its utility as a recombinant protein host.

### 3.3 Y‐7556, YB‐378, YB‐4289, and Y‐17741 possess thick cell walls relative to other strains

Since gene expression differed most among strains following growth with methanol (Condition C), we analyzed the relative expression of genes in the methanol utilization (Mut) pathway. Expression of Mut genes varied among strains with four dominant phenotypic patterns: (1) strong methanol oxidation and dihydroxyacetone (DHA) synthesis, (2) strong methanol oxidation but moderate DHA synthesis, (3) weak methanol oxidation but strong pentose phosphate pathway (PPP) activity, and (4) moderate methanol oxidation but weak DHA synthesis (Figure 2b). Mut genes were expressed weakly in Y‐7556, YB‐378, YB‐4289, and Y‐17741 relative to the other strains (Figure S3). Strong relative expression of *TKL1* in these strains may suggest an increased flux through the PPP to create intermediates for central metabolism (Feng, Liu, Weber, & Li, 2018; Krainer et al., 2012). Increased utilization of the PPP could explain in part how these strains still attain reasonable cell densities despite weak expression of Mut genes (Figure S1; Nocon et al., 2014). In addition, strong uptake of methanol requires high concomitant oxygen demand (Pelechano and Pérez‐Ortín, 2009), but expression of genes associated with hypoxia suggested no such oxygen demand in these strains (Figure S3; Baumann et al., 2011).

**Figure 3:**
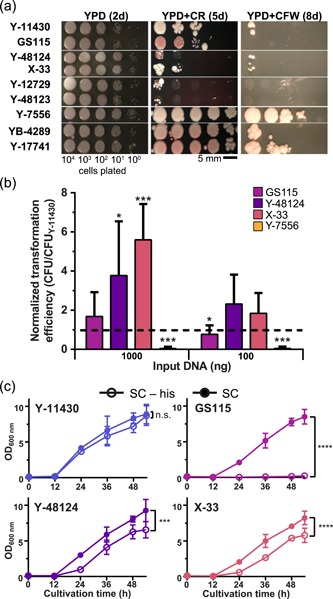
Phenotypic analysis of mutations affecting growth, cell wall composition, and DNA repair in select strains. (a) Plating of serial dilutions of strains on YPD and YPD supplemented with either Congo red or Calcofluor white dye. (b) Transformation efficiency of select strains relative to Y‐11430 for each of two input amounts of linearized plasmid DNA. Error bars represent standard deviation of six replicates. Significance was calculated using a one‐sample Student's *t* test relative to baseline =1. (c) Growth curves for each biotechnological strain in synthetic complete medium with histidine (SC) or without histidine (SC‐his). Error bars represent 95% confidence intervals. Significance was calculated using an extra sum‐of‐squares *F* test on nonlinear regressions for each culture. Regressions were created using a least‐squares fit to the Gompertz growth equation. *p ≤ .05; **p ≤ .01; ***p ≤ .001; ****p ≤ .0001; n.s., not significant [Color figure can be viewed at wileyonlinelibrary.com]

As a potential explanation for reduced expression of methanol utilization genes, we hypothesized that these strains did not uptake methanol efficiently. We previously had observed that the typical amount of zymolyase used to digest the cell wall for RNA extraction was insufficient in these strains (Figure S3). We thus hypothesized that the cell wall of these strains might be thicker, slowing the diffusion of methanol into the cell. Higher expression of key cell wall and glycosylation genes in these strains also supports our hypothesis of a thicker cell wall for these strains (Figure S3). We further observed strong expression of *YNL190W, TIP1, PIR1*, and *SNQ2*, which previously have correlated with resistance to organic solvents in *S. cerevisiae* (Nishida, Ozato, Matsui, Kuroda, & Ueda, 2013). To further test our hypothesis, we plated these and other strains on agar containing Congo red or Calcofluor white—known dyes for interrogating the integrity of cell walls (Ram & Klis, 2006). Each of these strains displayed complete resistance to Congo red and near complete resistance to Calcofluor white, unlike any of the other strains in this study (Figure 3a). Given that only these strains share the larger Rsf2p variant found in *S. cerevisiae*, it is possible that this TF influences alcohol metabolism and cellular morphogenesis through a structural alteration of the cell wall.

### 3.4 Thick cell wall leads to poor transformation efficiency in Y‐7556, YB‐378, YB‐4289, and Y‐17741

We next posited that a thicker cell wall would impede DNA uptake and lead to reduced transformation efficiencies. Y‐7556 and related strains did show extremely poor transformation efficiencies relative to Y‐11430 (Figure 3b). Repeated attempts to generate multi‐copy integrants expressing rhGH yielded fewer than 10 colonies for each of Y‐7556, YB‐378, YB‐4289, and Y‐17741 using common methods to prepare competent cells (Lin‐Cereghino et al., 2005; Wu & Letchworth, 2004). Confirmed expression of hGH was only achieved in YB‐4289 and Y‐17741, and these strains showed comparable or significantly weaker rhGH secretion relative to Y‐11430 (Figure S4). Due to weak expression of Mut pathway genes, a restrictive cell wall, and the difficulty in obtaining multi‐copy integrants, these four strains (Y‐7556, YB‐378, YB‐4289, or Y‐17741) do not offer significant advantages for recombinant protein production and we did not pursue them for further study.

### 3.5 Protein production is greater in Y‐11430 than Y‐12729 or Y‐48123

We observed comparable gene expression among Y‐11430, Y‐12729, and Y‐48123 across all conditions tested (Figure 2a; Figure S3), including expression of Mut genes (Figure 2b). We transformed all three strains to express secreted rhGH. Y‐11430 produced more protein than both Y‐12729 and Y‐48123 independent of copy number (Figure S4). None of our analyses revealed a significant metabolic, transformation efficiency, or productivity advantage in Y‐12729 and Y‐48123 relative to the genetically similar Y‐11430 strain.

### 3.6 Mutations in *HIS4* gene impair GS115 and X‐33 growth

Robust growth and high cell‐specific productivity are necessary to achieve high volumetric productivity. Surprisingly, GS115 grew slowly even in complex media containing histidine and to lower final cell densities than the other strains (Figure S1). We performed PCA on the gene expression data from Condition A (24 hr; glycerol) and found GS115 was distinct from the other strains by principal component two (Figure S5). This principal component was enriched for the amino acid metabolism gene set (ES = 1.89; *p* < .05; false discovery rate <10%) and genes associated with glutathione accumulation (*NIT1*) in response to osmotic stress (*CCC2, PDR12, ZPS1, FRE1, CTR1*; Table S4). Previous work in *S. cerevisiae* has shown a pH‐dependent, toxic sensitivity to metal salts caused by histidine auxotrophy, suggesting a role for histidine in mitigating osmotic stress (Pearce & Sherman, 1999). Analysis of gene expression under Conditions B and C also indicated differences in amino acid metabolism between GS115 and the other strains (Table S3; Table S5). This collective transcriptomic signature may suggest that, although there was available histidine in the media for GS115 during the first 24 hr of growth, a more permeable cell wall or membrane is unable to mitigate the osmotic imbalance already exacerbated by a lack of histidine inside the cell. Under Conditions B and C, however, GS115 likely depleted any histidine present in the medium.

Given the highly conserved nature of position 557 in His4p, we hypothesized that histidine synthesis may be impaired in Y‐48124 and X‐33, which contain substitutions at this position in His4p like GS115. We evaluated these strains for histidine auxotrophy or bradytrophy (slowed growth) by comparing growth of Y‐11430, GS115, Y‐48124, and X‐33 in both SC and SC‐his dropout media (Figure 3c). Surprisingly, we observed that the Y‐48124 and X‐33 strains reported to have a wild‐type phenotype (Higgins et al., 1998) also grew slowly without histidine. This slowed growth was most dramatic in the first 24 hr (*p* < .0001); growth over the entire cultivation in SC‐his media (54 hr) was, however, still significantly lower than in SC media (Y‐48124, *p* < .001; X‐33, *p* < .0001). Thus, both Y‐48124 and X‐33 are indeed bradytrophic for histidine, confirming the predicted functional impact of the p.C557G missense mutation determined in *HIS4*.

### 3.7 Cell wall defects in Y‐48124 and X‐33 lead to enhanced transformation efficiencies

Since GS115, Y‐48124, and X‐33 share mutations in several cell wall genes, we interrogated gene expression in these strains under Condition A, when biogenesis of the cell wall is most active. We analyzed the leading edge of GSEA results for cell wall and sporulation gene sets and identified a set of cell wall genes that distinguished these strains from the others (Figure S6). In particular, upregulated expression of *GAS2, CWP1, YPS1, TIP1, SPS22, SSP2*, and *PTP2* suggests strong induction of the cell wall integrity (CWI) pathway (Rodríguez‐Peña, García, Nombela, & Arroyo, 2010). Given this gene signature correlates with observed osmotic sensitivity in GS115, strong CWI induction suggests a weakened cell wall in Y‐48124 and X‐33.

To validate the transcriptomic evidence of altered cell walls in the family of GS115 strains, we compared growth of these strains to the other variants in the presence of Congo red or Calcofluor white (Figure 3a). While Y‐11430 excluded the Congo red dye (white colonies), GS115 incorporated it (red colonies), indicating a difference in cell wall biogenesis. Y‐48124 and X‐33 were more sensitive to Congo red than Y‐11430 and GS115, perhaps indicating a more permeable cell wall. In the presence of Calcofluor white, a few Y‐48124 and X‐33 colonies were observed after 8 days of growth, unlike GS115, suggesting a difference in cell wall composition (Ram & Klis, 2006).

We believed that a more permeable cell wall could enhance transformation efficiency, so we transformed two different amounts of three linearized plasmids targeting the *AOX1* locus into GS115, Y‐48124, and X‐33 and compared the efficiency to Y‐11430. We observed GS115 to display a similar transformation efficiency as Y‐11430. Y‐48124, which has the *RCR2* mutation, showed a near four‐fold improvement and X‐33, which possesses both the *RCR2* and *RVB1* mutations, showed a near six‐fold improvement over Y‐11430. These observed trends in transformation efficiency agreed with the Congo red and Calcofluor white permeability assays, further suggesting that the cell walls of Y‐48124 and X‐33 are more permissive than Y‐11430 to both small molecules and DNA.

### 3.8 Productivity comparison among strains

**Figure 4:**
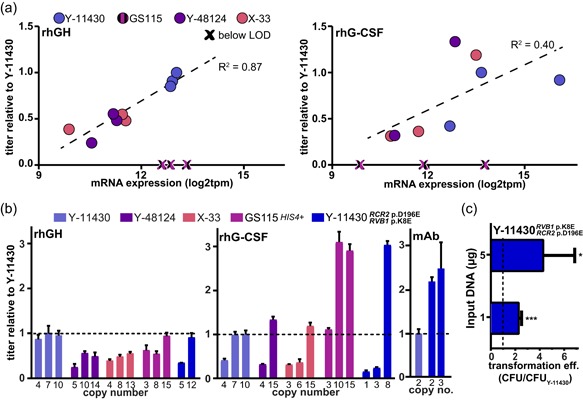
Productivity of strains expressing a hormone (rhGH), cytokine (rhG‐CSF), or monoclonal antibody (trastuzumab). (a) Secreted protein titer (relative to highest‐secreting Y‐11430 strain) versus transgene messenger RNA expression for *K. phaffii* strains. Titers from GS115 strains were negligible relative to Y‐11430 (noted with an “X”). Linear fit between protein titer and transgene expression is depicted. (b) Relative titer for biotechnological and engineered strains expressing rhGH, rhG‐CSF, or trastuzumab. Titer shown relative to the highest producing Y‐11430 clone, as indicated by the dashed line. The copy number of each strain is indicated. (c) Transformation efficiency of Y‐11430 RCR2^D196E^ RVB1^K8E^ relative to Y‐11430 for each of two input amounts of various linearized plasmids. Error bars represent standard deviation of four and six replicates for 5 and 1‐µg inputs, respectively. Significance was calculated using a one‐sample Student's *t* test relative to baseline =1. *p ≤ .05; ***p ≤ .001 [Color figure can be viewed at wileyonlinelibrary.com]

Following the transcriptomic evaluation of each strain and targeted phenotypic assays, we deprioritized several strains for routine use as an optimal base strain of *K. phaffii* or for future engineering. We had difficulty obtaining multi‐copy integrants in Y‐7556, YB‐378, YB‐4289, and Y‐17741, owing to a thicker cell wall, and expression from successful integrants was relatively weak. Y‐12729 and Y‐48123 offered no significant benefit to overcome the current widespread use of the genetically similar Y‐11430. Therefore, we evaluated the productivity of the remaining strains (Y‐11430, GS115, Y‐48124, X‐33) by transforming all four strains to express secreted rhGH or rhG‐CSF under control of the *AOX1* promoter at the *AOX1* locus. We characterized three clones of each strain ranging from low to high copy number for growth, transgene expression, secreted protein titer, and associated transcriptomic signature by RNA‐Seq (Figure 4).

### 3.9 Strong transgene expression of rhGH correlates with strong secretion in Y‐11430

Since rhGH is a small protein secreted with relative efficiency, we did not expect differences in the cell wall manifest among strains to greatly impact productivity. Surprisingly, however, Y‐11430 produced the most rhGH due to two‐fold higher transgene expression (*p*‐adj < 10^−5^) that could not be explained by differences in copy number (Figure 4). A similar strength of rhGH transcription was seen in GS115, but low cell density precluded appreciable titers (Figure S7). No gene sets were significantly enriched in Y‐11430 versus Y‐48124 and X‐33 (Table S6). Cell‐wide transcriptional activity was not higher in Y‐11430 relative to the other strains, as revealed by differential expression analysis, indicating only transgene expression was higher. To investigate altered regulation of the *AOX1* promoter as a possible explanation, we compared native expression of *AOX1* between strains, but found no significant difference. Interestingly, the seven most DEGs between Y‐11430 and Y‐48124/X‐33 are located on the killer plasmids no longer maintained in the latter strains. Given that these plasmids express replication and transcription genes for self‐maintenance (Love et al., 2016; Sturmberger et al., 2016), benefits for transgene expression could be correlated, but further study is necessary.

### 3.10 Permeability of Y‐48124 and X‐33 cell walls permit increased rhG‐CSF secretion

Unlike rhGH, rhG‐CSF is known to form protein aggregates, and cultivation with surfactants helps dissociate protein bound to the cell wall of expressing cells (Bahrami et al., 2009). Expression of rhG‐CSF served as a good model to assess the effects on protein secretion of mutations in membrane‐sorting and cell wall proteins. As with rhGH, we observed higher transcription of rhG‐CSF in Y‐11430, but not GS115, relative to the other strains (*p*‐adj < .005). Despite strong transcription, volumetric productivity of rhG‐CSF in Y‐11430 was lower than in Y‐48124 and X‐33, which we attribute to a more permeable cell wall and membrane in the latter strains. A high copy number of transgene was required in Y‐48124 and X‐33 to achieve the requisite transcription of rhG‐CSF needed for high productivity (Figure 4a). No gene sets enriched in Y‐11430 versus Y‐48124 or X‐33 were detected with high confidence (Table S6), and the *GAS1* and *GAS2* cell wall genes were the only non‐killer plasmid genes significantly differentially expressed between these two groups across both rhGH and rhG‐CSF production. Expression of *GAS2* was two‐ to three‐fold higher in Y‐48124 and X‐33 relative to Y‐11430, while expression of *GAS1* was two‐ to four‐fold lower. *GAS1* knockouts have been previously shown to enhance release of membrane‐associated proteins (Marx et al., 2006), providing further support for our hypothesis that the GS115 family has a more permeable cell membrane and wall.

### 3.11 Complementation of GS115 with *HIS4* restores high productivity

We observed that GS115 had similar levels of rhGH and rhG‐CSF transcription as Y‐11430, but challenges from histidine auxotrophy resulted in poor growth of all heterologous protein‐expressing GS115 strains (Figure S7). We therefore complemented GS115 with the wild‐type *HIS4* gene and tested production of rhGH and rhG‐CSF in this strain. The GS115 *HIS4* ^+^ strain produced rhGH titers comparable to Y‐11430, but only at high transgene copy number (>8) unlike with Y‐11430 (Figure 4b). Similarly, only high transgene copy number yielded the highest rhG‐CSF titers, but these titers were nearly three times higher in GS115 *HIS4* ^+^ than the best Y‐11430, Y‐48124, or X‐33 clones. These exceptional results may be explained by GS115 *HIS4* ^+^ possessing a permeable cell wall similar to Y‐48124 and X‐33, but with nearly as strong transcription of rhG‐CSF as Y‐11430. Despite these benefits, this modified version of GS115 may present other challenges for routine use in bioprocesses and large‐scale fermentation. Performance for these applications may be impaired by other mutations harbored among the 73 additional SNPs that reside in genes related to osmotic resistance and growth and show altered profiles of expression (as revealed by our transcriptomic analysis).

### 3.12 Engineered Y‐11430 variant offers optimal titers and enhanced transformation efficiency

One practical application for the broad comparative analysis of different banked strains of an organism we have presented here is to guide targeted engineering of a base strain of choice to confer specific beneficial advantages while avoiding other potential deleterious or disadvantaged variants. To this end, we aimed to generate an engineered variant of Y‐11430, which strongly expressed recombinant genes and Mut genes and has no mutational background, to include the enhanced transformation efficiency and cell wall permeability found in X‐33. From our genomic and transcriptomic analyses, we hypothesized that the mutated variants of *RCR2* (vacuolar sorting of membrane‐bound proteins) and *RVB1* (DNA repair) present in X‐33, but not GS115, contributed most to these traits. We, therefore, introduced the *RCR2* p.D196E and *RVB1* p.K8E variants into Y‐11430 and then transformed rhGH, rhG‐CSF, and trastuzumab expression cassettes into this strain. While these two mutations alone did not recover the full enhancement of transformation efficiency observed in X‐33, the efficiency did improve up to four‐fold relative to Y‐11430 (Figure 4c). This result may suggest that additional mutations from X‐33, such as in DNA repair genes *RAD5, EXO1*, and *DNL4*, could provide an additional boost to transformation efficiency, though perhaps with reduced genomic stability. As with the GS115 *HIS4* ^+^ strain, rhGH titers achieved using the Y‐11430 *RCR2* p.D196E+*RVB1* p.K8E strain were comparable to Y‐11430 at high copy number (Figure 4b). Interestingly, secreted rhG‐CSF titers were nearly three‐fold higher than Y‐11430, Y‐48124, or X‐33 and secreted trastuzumab titers were more than two‐fold higher than Y‐11430 (Figure 4b, Figure S8). Collectively, these results suggest our engineered Y‐11430 variant successfully combines the strong transcriptional strength of Y‐11430 (high rhGH titers) with the cell wall permeability of X‐33 (high rhG‐CSF and trastuzumab titers) in a background free from uncharacterized mutations. Further targeted engineering of such a strain with known functional variants or genomic deletions should provide a rational path towards engineered enhanced strains for expression of recombinant proteins.

## 4 CONCLUSIONS

Here we have demonstrated an approach whereby easily acquired genomic and transcriptomic data on variants of a given organism enable prediction of performance that can guide selection of an optimal host. To date, the varied relative performance of *P. pastoris* strains has been reported with respect to product quality, titer, and growth (Blanchard et al., 2008; Huang et al., 2014; Razaghi et al., 2017). Our analyses revealed the integrity of the cell wall likely confers the largest source of variation in performance among these strains. We have demonstrated with transcriptomic analyses that the cell wall and membrane can limit carbon source uptake (e.g., Y‐7556), amino acid availability (e.g., X‐33), and resistance to osmotic stress (e.g., GS115). The cell wall and membrane also mediate the uptake of DNA, resulting in drastically different transformation efficiencies between strains with thick cell walls (e.g., Y‐7556) and those with more permissive cell walls (e.g., X‐33). We have also shown that strains with permeable cell walls can secrete membrane‐associated proteins more efficiently than others, as with rhG‐CSF expression in Y‐48124 and X‐33. With a systematic approach to understanding strain performance, we analyzed and conferred two beneficial features from the various base strains into an optimized strain, Y‐11430 *RCR2* p.D196E+*RVB1* p.K8E. This engineered base strain provided the best of transgene expression, robust growth, enhanced transformation efficiency, and improved secretion for high volumetric productivity of rhG‐CSF and trastuzumab relative to the baseline strain.

For *P. pastoris* or any recombinant host, standardization within the community around a single base strain should streamline and accelerate the optimization of the host and promote widespread adoption. Through an evidence‐based evaluation of *K. phaffii* base strains, we have provided clarity and predictability to the use of this host. Further, we have demonstrated that genome‐wide assays such as whole genome sequencing and RNA‐Seq afford an invaluable level of biological understanding, suggesting that additional probes of epigenetics, translation, or metabolomics may further elucidate biological mechanisms. Our data‐driven approach should be applicable to other organisms to enable identification of an optimal host. Collectively, these genome‐wide assays will form an essential toolbox for future efforts in strain engineering, and permit the rational design of a set of hosts optimized for diverse modalities of biologics or enzymes.

## Supporting information

Supplementary information
