# Supplementary material for: Comparative genome‐scale analysis of Pichia pastoris variants informs selection of an optimal base strain
Source: Biotechnol Bioeng. 2019 Nov 12;117(2):543–55. doi: 10.1002/bit.27209 (PMC7003935; doi:10.1002/bit.27209)
Supplement: Supplementary file 1 — Supplementary information [file BIT-117-543-s001.pdf]

# Comparative genome-scale analysis of *Pichia pastoris* variants informs selection of an optimal base strain

Joseph R. Brady[1,2], Charles A. Whittaker[1], Melody C. Tan[1], D. Lee Kristensen II[1], Duanduan Ma[1], Neil C. Dalvie[1,2], Kerry Routenberg Love[1], J. Christopher Love[1,2]†

1. Koch Institute for Integrative Cancer Research, Massachusetts Institute of Technology, Cambridge, MA
2. Department of Chemical Engineering, Massachusetts Institute of Technology, Cambridge, MA
† Corresponding author:
J. Christopher Love
77 Massachusetts Avenue / 76-253
Cambridge, MA 02139
United States
phone: (617) 324-2300
email: clove@mit.edu

## SUPPORTING INFORMATION

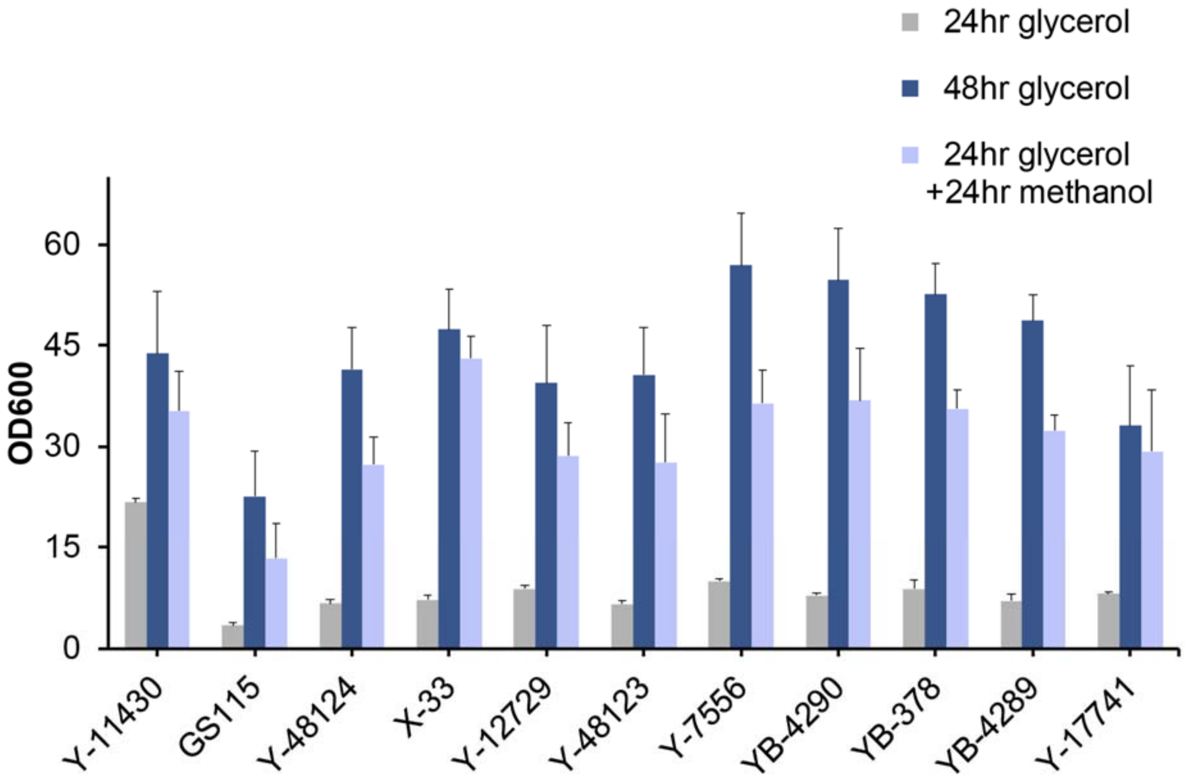


**Figure S1.** Growth of non-transgenic strains in complex glycerol-containing and methanol-containing media. Error bars represent the standard deviation of eight independent replicates (24hr glycerol condition) or four independent replicates (48hr glycerol and 24hr glycerol + 24hr methanol conditions).

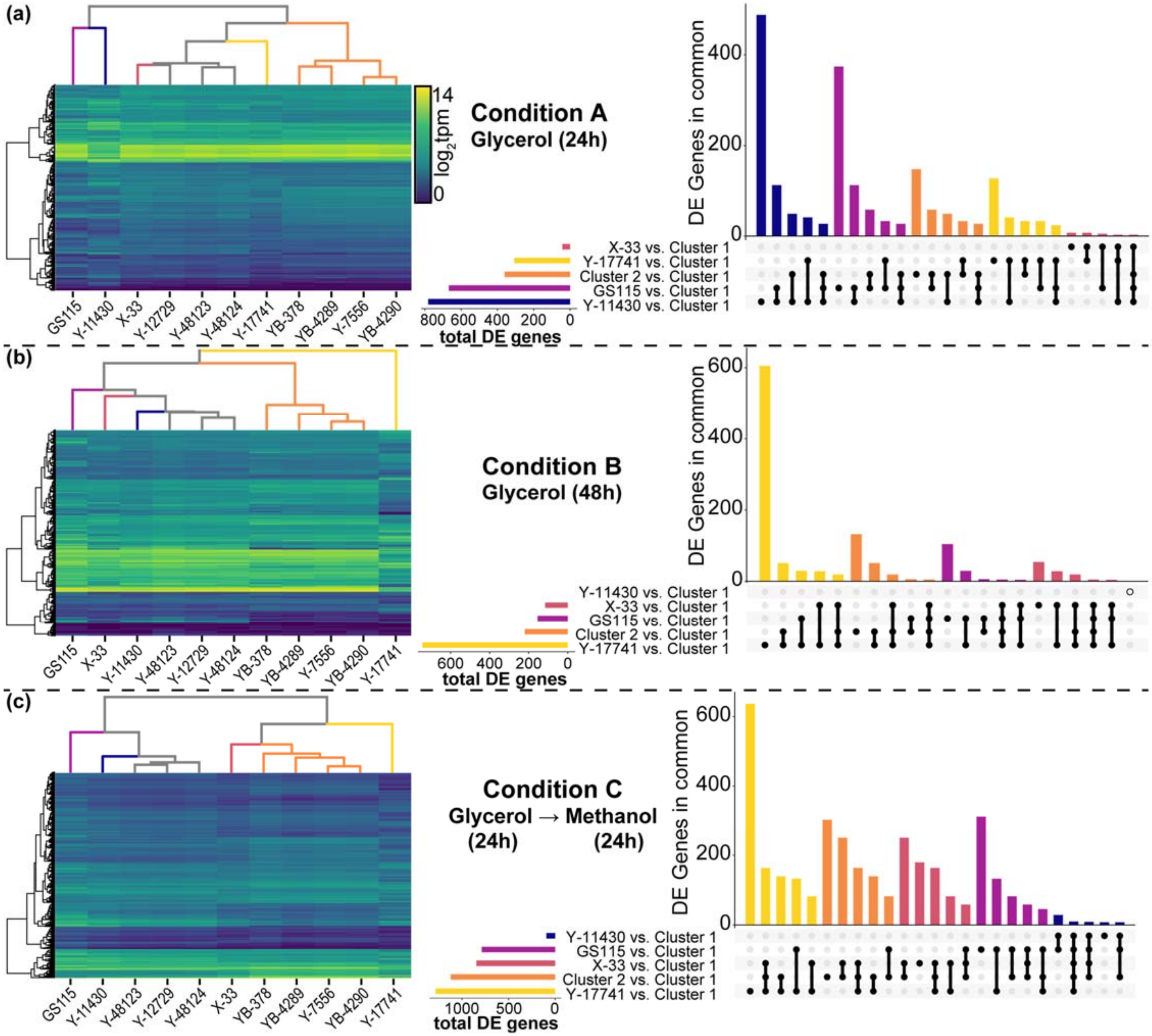


**Figure S2.** Visualization by heatmap and UpSet plot of differentially expressed genes in each condition. Gene expression heatmaps of all differentially expressed (DE) genes in any comparison within a given condition (a), (b), or (c). Upset plots show counts of DE genes for each phenotypic cluster. Vertical bars represent counts of DE gene intersections between the five phenotypic clusters. Clusters were determined by hierarchical clustering of DE genes on left, and colored accordingly with Cluster 1 in gray. All DE gene counts shown are relative to Cluster 1. Cluster 1 includes Y-48124, Y-12729, and Y-48123; Cluster 2 includes Y-7556/YB-4290, YB-378, and YB-4289.

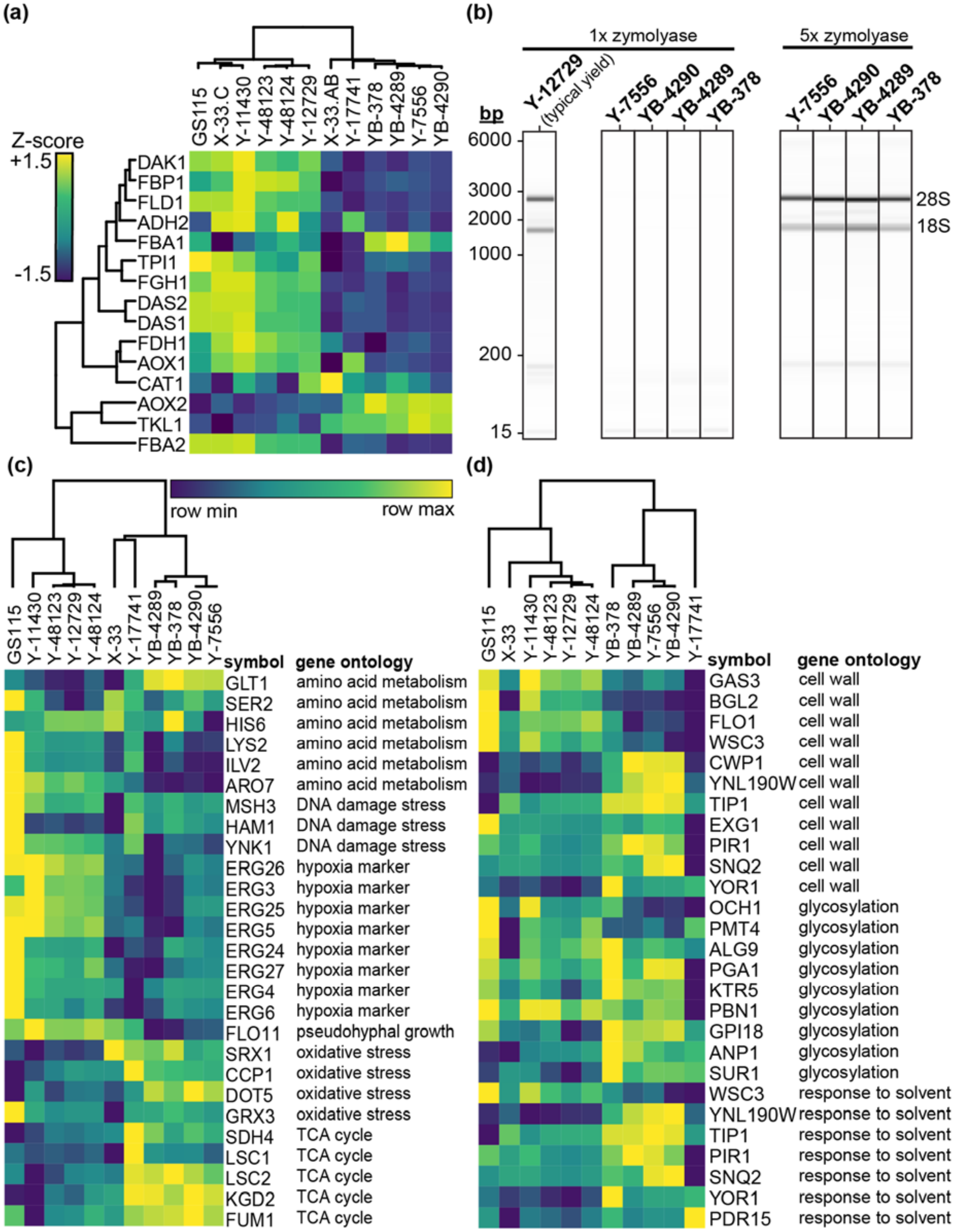


**Figure S3.** Expression of mut and cell wall integrity genes under 24hr glycerol + 24hr methanol condition. (a) Expression of mut genes for all strains. (b) Automated electrophoresis of purified total RNA for Cluster 2 strains compared to a control strain, Y-12729. The relative amount of cell wall digestive enzyme, zymolyase, is depicted as 1x or 5x excess enzyme. (c) Expression of genes associated with response to oxygen availability. (d) Expression of genes associated with cell wall integrity.

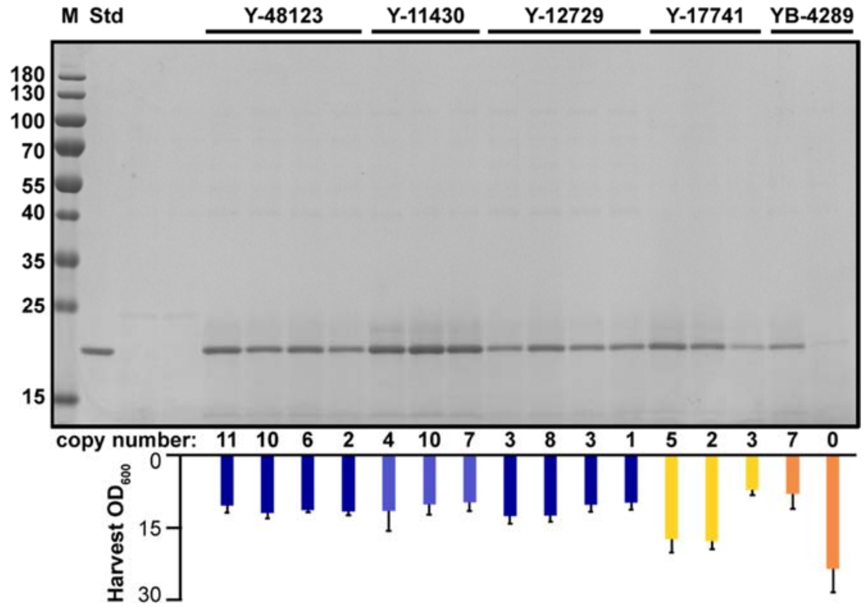


**Figure S4.** SDS-PAGE gel showing expression of hGH by select strains. Gels were loaded with 15 µL of supernatant and stained with Coomassie blue. Reference standard concentration was 0.1 mg/mL. M – molecular weight marker. The copy number and harvest $OD_{600}$ for each clone are shown below the respective lane. Error bars represent the standard deviation in four independent measurements.

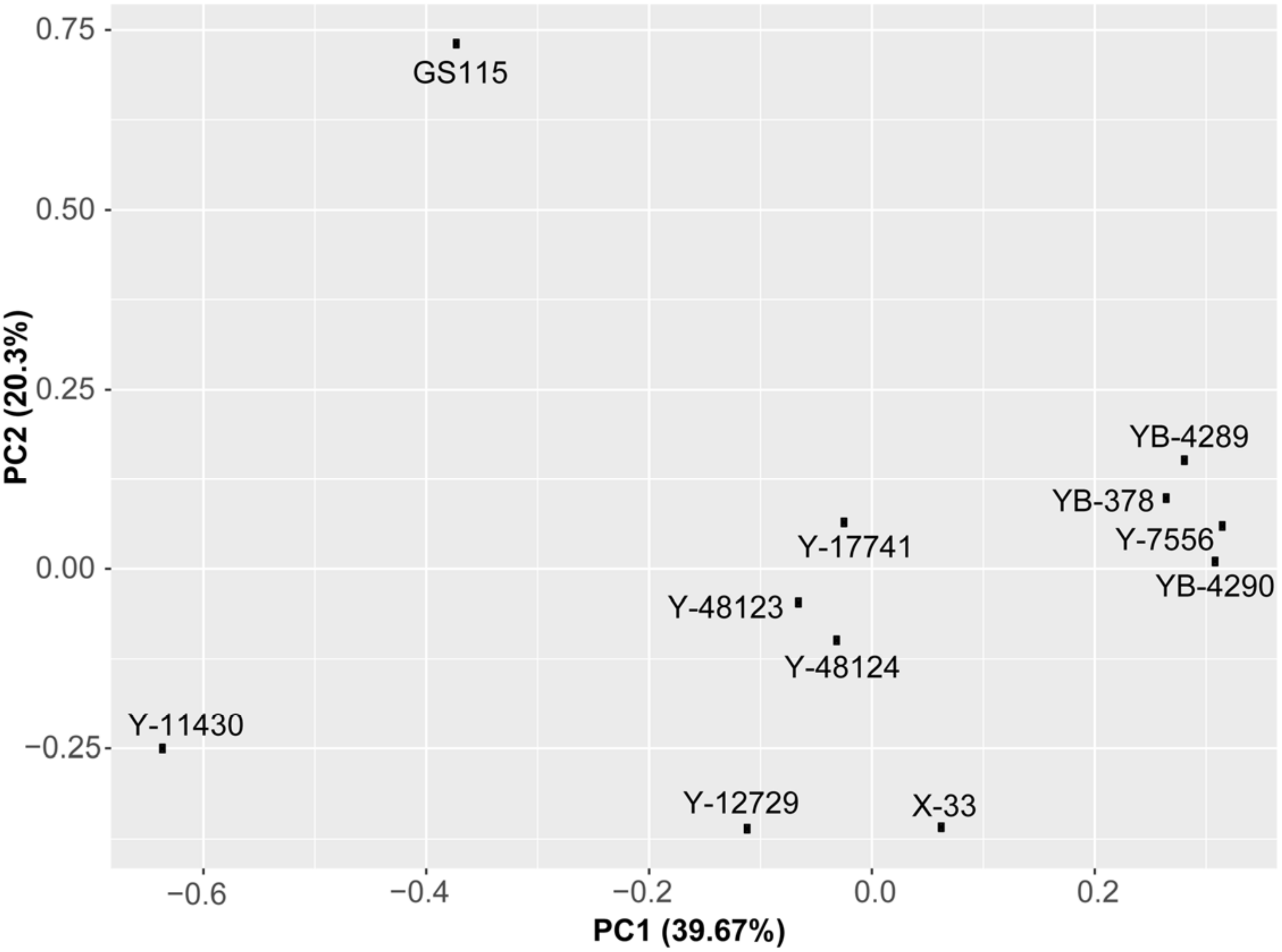


**Figure S5.** Principal component analysis biplot for all strains under Condition A (24h glycerol).

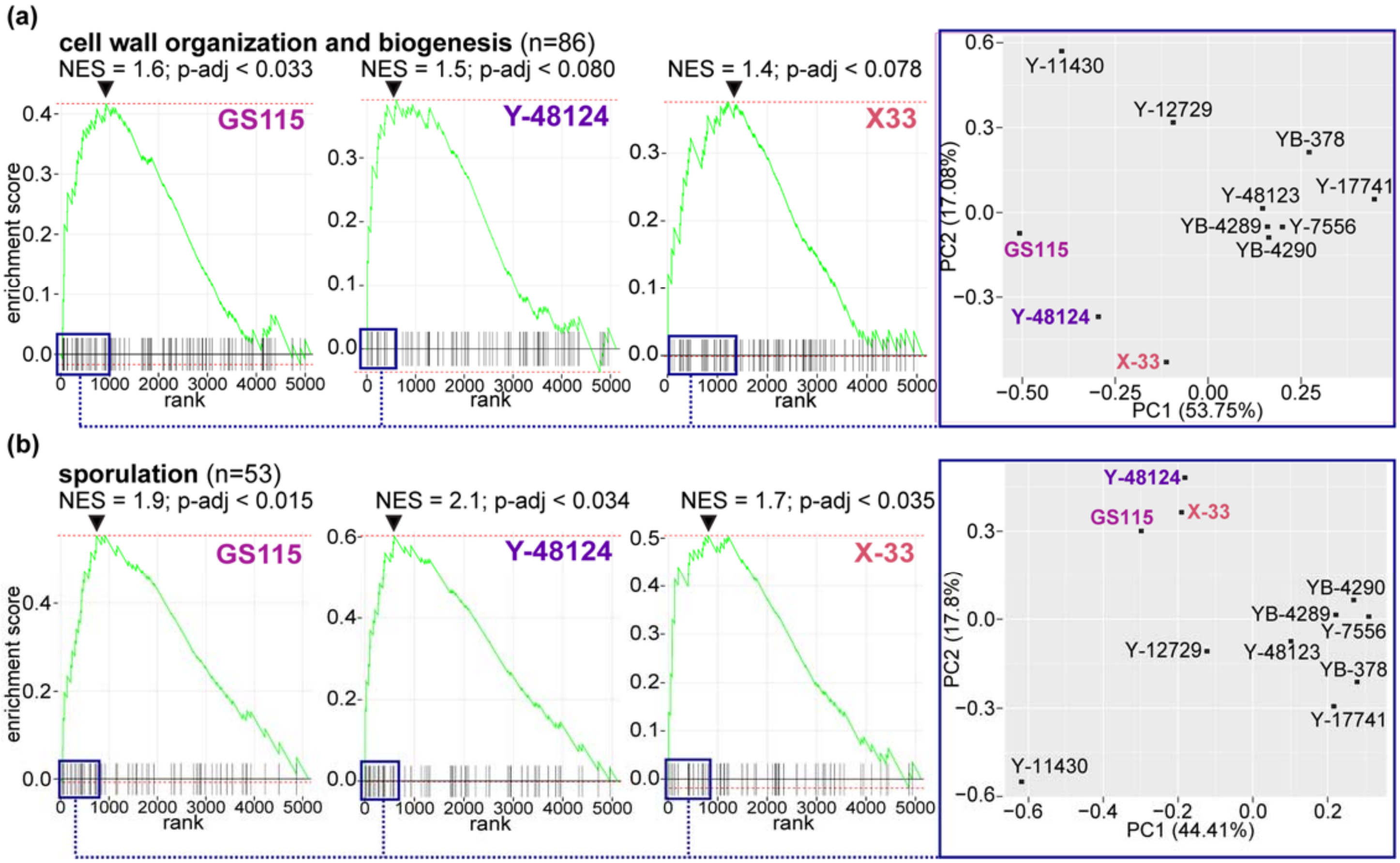


**Figure S6.** Leading edge analysis of gene-set enrichment analysis (GSEA) results for cell wall and sporulation genes in the GS115 family. (a) GSEA enrichment plot for the cell wall organization and biogenesis gene set measured under Condition A (24h glycerol). Normalized enrichment score (NES) and Benjamini-Hochberg adjusted *P* value are denoted for each strain; all comparisons were significant ($P < 0.05$, FDR < 10%). Principal component analysis plot using only leading edge genes is shown at right. (b) GSEA enrichment and principal component analysis plots for the sporulation gene set under Condition A.

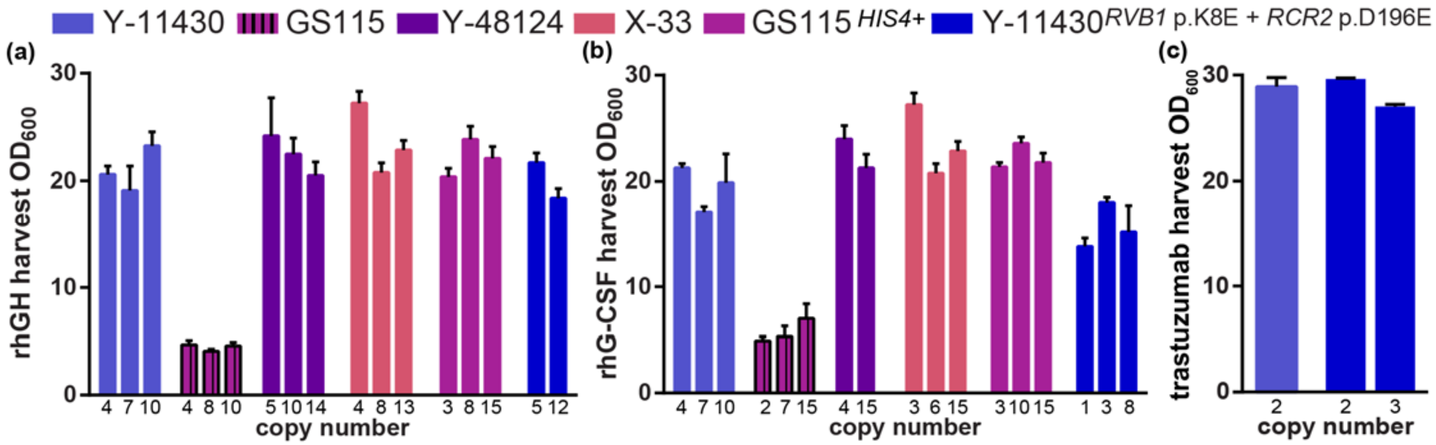


**Figure S7.** Growth of (a) hGH-expressing, (b) G-CSF-expressing, or (c) trastuzumab-expressing strains after 24h growth with glycerol followed by 24h growth with methanol. Error bars represent the standard deviation of three independent replicates of each clone. The copy number of each rhGH, rhG-CSF, or trastuzumab clone, rounded to the nearest integer, is listed.

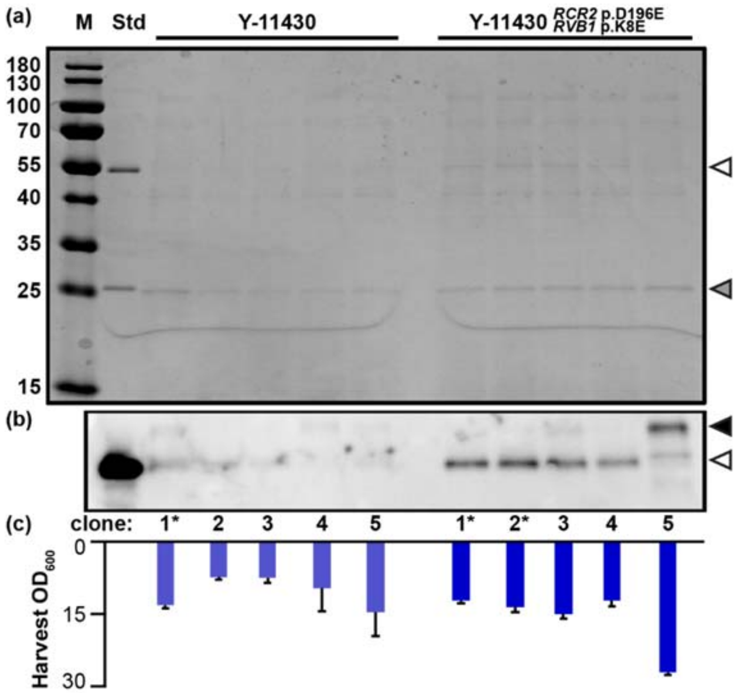


**Figure S8.** Expression testing of trastuzumab-expressing clones for both Y-11430 and Y-11430 *RCR2* p.D196E + *RVB1* p.K8E. (a) SDS-PAGE gel showing trastuzumab expression of ten clones. Gels were loaded with 15 µL of supernatant and stained with Coomassie blue. Concentration of Human IgG1 reference standard was 0.05 mg/mL. M – molecular weight marker. White triangle indicates the expected position of the heavy chain fragment. Gray triangle indicates the expected position of the light chain fragment. (b) Western blot for human IgG1 Fc fragment (heavy chain). Black triangle indicates the expected position of glycosylated heavy chain. (c) The clone number and harvest $OD_{600}$ for each clone are shown. Error bars represent the standard deviation in four independent measurements. Clones marked with an asterisk were further analyzed for copy number and secreted titer by ELISA following cultivation in triplicate.

**Table S1**. Recombinant gene sequences

| Gene name | Nucleotide sequence |
|---|---|
| rhGH | TTCCCAACTATCCCATTGTCCAGATTGTTCGACAACGCTATGTTGAGAGCTCACAGATTGCACCAGTTGGCTTT<br>CGACACTTACCAAGAGTTCGAAGAGGCTTACATCCCAAAAGAGCAGAAGTACTCCTTCTTGCAAAACCCTCAGA<br>CTTCCTTGTGTTTCTCCGAGTCCATTCCAACTCCATCCAACAGAGAAGAGACTCAGCAGAAGTCCAACTTGGAG<br>TTGTTGAGAATCTCCTTGTTGTTGATCCAGTCCTGGTTGGAGCCAGTTCAGTTCTTGAGATCCGTTTTCGCTAA<br>CTCCTTGGTTTACGGTGCTTCCGACTCTAACGTTTACGACTTGTTGAAGGACTTGGAAGAGGGTATCCAGACTT<br>TGATGGGTAGATTGGAAGATGGTTCCCCAAGAACTGGTCAGATCTTCAAGCAGACTTACTCTAAGTTCGACACT<br>AACTCCCACAACGACGACGCTTTGTTGAAGAACTACGGTTTGTTGTACTGTTTCAGAAAGGACATGGACAAGGT<br>TGAGACTTTCTTGAGAATCGTTCAGTGTAGATCCGTTGAGGGTTCCTGTGGTTTCTAA |
| rG-CSF | ATGACTCCTTTGGGTCCAGCTTCTTCCTTGCCTCAATCCTTCTTGTTGAAGTGTTTGGAGCAGGTTAGAAAGAT<br>CCAGGGTGATGGTGCTGCTTTGCAAGAGAAGTTGTGTGCTACTTACAAGTTGTGTCACCCAGAAGAGTTGGTTT<br>TGTTGGGTCACTCCTTGGGTATTCCTTGGGCTCCATTGTCCTCTTGTCCATCCCAAGCTTTGCAATTGGCTGGT<br>TGTTTGTCCCAATTGCACTCCGGTTTGTTCTTGTACCAGGGTTTGTTGCAAGCTTTGGAGGGTATTTCTCCAGA<br>GTTGGGTCCAACTTTGGACACATTGCAGTTGGACGTTGCTGACTTCGCTACTACTATCTGGCAACAGATGGAAG<br>AATTGGGTATGGCTCCAGCTTTGCAGCCAACTCAAGGTGCTATGCCAGCTTTTGCTTCTGCTTTCCAGAGAAGA<br>GCTGGTGGTGTTTTGGTTGCTTCTCACTTGCAGTCTTTCTTGGAGGTTTCCTACAGAGTTTTGAGACACTTGGC<br>TCAACCATAA |
| rIFNα-2b | TGTGACTTGCCTCAAACTCACTCCCTGGGTTCTAGAAGAACCTTGATGTTGTTGGCCCAGATGAGAAGAATCTC<br>CTTGTTCTCCTGCCTGAAGGACAGACACGATTTCGGTTTCCCACAAGAAGAGTTCGGTAACCAGTTCCAGAAGG<br>CTGAGACTATTCCAGTCTTGCACGAGATGATCCAGCAGATCTTCAACCTGTTCTCCACTAAGGATTCTTCCGCT<br>GCTTGGGACGAAACCTTGTTGGACAAGTTCTACACCGAGTTGTACCAGCAGTTGAACGACTTGGAGGCCTGTGT<br>TATTCAAGGTGTTGGTGTTACCGAGACTCCACTGATGAAGGAGGACTCCATTTTGGCCGTCAGAAAGTACTTCC<br>AGAGAATCACCCTGTACCTGAAAGAGAAGAAGTACTCTCCTTGCGCCTGGGAAGTTGTTAGAGCTGAGATTATG<br>AGATCCTTCTCCTTGTCCACCAACCTGCAAGAGTCCTTGAGATCCAAAGAGTAA |
| trastuzumab | >heavy chain<br>GAGGTTCAATTGGTTGAATCCGGTGGTGGTTTGGTTCAACCAGGTGGTTCTTTGAGATTGTCCTGTGCTGCTTCC<br>GGTTTCAACATCAAGGACACTTACATCCACTGGGTTAGACAGGCTCCAGGTAAAGGTTTGGAATGGGTTGCTAGA<br>ATCTACCCCACTAACGGTTACACTAGATACGCCGACTCTGTCAAGGGAAGATTCACTATTTCTGCCGACACCTCCA<br>AGAACACTGCCTACTTGCAAATGAACTCCTTGAGAGCTGAGGACACCGCCGTTTACTACTGTTCTAGATGGGGTG<br>GTGATGGTTTCTACGCTATGGATTACTGGGGTCAGGGTACTTTGGTTACCGTTTCTTCTGCTTCCACTAAGGGTCC<br>ATCTGTTTTTCCATTGGCTCCCTCATCTAAGTCCACTTCCGGTGGTACTGCTGCTTTGGGTTGTTTGGTTAAGGAC<br>TACTTCCCAGAGCCAGTTACTGTTTCCTGGAACTCTGGTGCTTTGACTTCTGGTGTTCACACTTTCCCAGCTGTCT<br>TGCAATCTTCTGGTCTGTACTCTTTGTCCTCCGTCGTTACTGTTCCTTCTTCCTCCTTGGGTACTCAAACCTACATC<br>TGCAACGTCAACCACAAGCCATCCAACACCAAGGTTGACAAGAAGGTTGAGCCAAAGTCCTGTGACAAGACTCAC<br>ACTTGTCCACCATGTCCAGCTCCAGAATTACTTGGTGGTCCTTCCGTTTTCTTGTTCCCACCAAAGCCAAAGGACA<br>CCCTGATGATTTCTAGAACCCCAGAGGTTACCTGTGTCGTTGTTGACGTTTCTCACGAGGACCCTGAGGTTAAGT<br>TCAACTGGTACGTTGACGGTGTTGAGGTTCACAACGCTAAGACTAAGCCTAGAGAAGAACAGTACAACTCCACCT<br>ACAGAGTTGTCTCCGTTTTGACTGTGTTGCACCAGGATTGGCTGAACGGTAAAGAATACAAGTGCAAGGTGTCCA<br>ACAAGGCTTTGCCAGCTCCAATCGAAAAGACTATCTCCAAGGCTAAGGGTCAGCCAAGAGAGCCACAAGTTTACA<br>CTTTGCCACCATCCAGAGAgGAGaTGACCAAGAACCAGGTTTCCTTGACCTGTTTGGTCAAGGGTTTCTACCCATC<br>CGACATTGCTGTTGAATGGGAGTCTAACGGTCAGCCTGAGAACAACTACAAGACTACCCCACCAGTTTTGGACTC<br>TGACGGTTCATTCTTCCTGTACTCCAAGTTGACCGTCGACAAGTCCAGATGGCAACAGGGTAACGTTTTCTCCTGT<br>TCCGTTATGCATGAGGCCTTGCACAACCACTACACTCAAAAGTCCTTGTCTCTGTCCCCTGGTAAGTAA<br>>light chain<br>GACATCCAAATGACTCAATCTCCCTCTTCCTTGTCCGCTTCTGTTGGTGACAGAGTTACCATCACTTGTAGAGCTT<br>CCCAGGACGTTAACACTGCTGTTGCTTGGTATCAGCAGAAGCCAGGTAAGGCTCCAAAGTTGTTGATCTACTCCG<br>CCTCCTTCTTGTACTCTGGTGTTCCATCTAGATTCTCCGGTTCCAGATCCGGTACTGACTTTACCTTGACCATCTC<br>CTCATTGCAGCCAGAGGATTTCGCTACCTACTACTGTCAACAGCACTACACTACTCCACCAACTTTCGGTCAGGG<br>TACTAAGGTTGAGATTAAGAGAACTGTTGCTGCTCCATCCGTGTTCATTTTCCCACCATCTGACGAGCAACTGAAG<br>TCTGGTACTGCTTCCGTTGTCTGCTTGCTGAACAACTTCTACCCAAGAGAAGCCAAGGTTCAGTGGAAGGTTGAT<br>AACGCCTTGCAATCCGGTAACTCCCAAGAATCCGTTACTGAGCAAGACTCCAAGGACTCCACTTACTCATTGTCCT<br>CCACCTTGACTTTGTCCAAGGCCGATTACGAAAAGCACAAGGTTTACGCCTGTGAGGTTACTCACCAAGGTTTGT<br>CCTCTCCAGTTACCAAGTCCTTCAACAGAGGTGAGTGTTAA |

**Table S2**. Primer sequences for Sanger sequencing and qPCR

| | Target | Fwd primer sequence | Rev primer sequence |
|---|---|---|---|
| Sanger | chr1_1703668_T_G (X-33/Y-48124) | GAGAAAGTTGGCCTCTATATTCC | CTTCTGGGGTAAGGGTACC |
| Sanger | chr2_1805135_G_A (Y-12729) | CCCTGTATTACTATCTAAAACTG TTAGAG | GCCTGCGCGTAGATATTC |
| Sanger | chr3_1072726_T_C (Y-7556/YB-4290) | TATGCACGTTAGGAAGAAGATG | AATCAAACAATTGGATAAA GACCC |
| Sanger | chr3_1777595_G_C (Y-48123) | ATGTTCTCGTTCTCCATTTCTC | CTGCCAAATCAAAAGGTCT TAG |
| Sanger | chr4_969066_A_G (X-33) | ACCTTACGAGCTTCATGG | GCTTGGCCTACAAAACC |
| qPCR | hGH (qPCR amplicon) | ACCAGTTGGCTTTCGACAC | TGGAATGGACTCGGAGAAAC |
| qPCR | G-CSF (qPCR amplicon) | AAAGATCCAGGGTGATGGTG | CTTGGGATGGACAAGAGGAC |
| qPCR | β -actin (qPCR amplicon) | ATTGCTGAGCGTATGCAA | CCACCGATCCATACGGAGTA CT |
| qPCR | trastuzumab HC (qPCR amplicon) | TGGCTCCCTCATCTAAGTCC | GCTGGGAAAGTGTGAACACC |
| qPCR | trastuzumab LC (qPCR amplicon) | GAACTGTTGCTGCTCCATCC | CGTTATCAACCTTCCACTGAACC |
| qPCR | hGH ORF (qPCR standard) | ATGAGATTTCCTTCAATTTTTAC TGCT | TTAGAAACCACAGGAACCC |
| qPCR | G-CSF ORF (qPCR standard) | ATGAGATTTCCTTCAATTTTTAC TGCT | TGGTTGAGCCAAGTGTCT |
| qPCR | β-actin ORF (qPCR standard) | CGGTATGTGTAAGGCCGGAT | GAGGTGCACAATGGATGGTC |
| qPCR | trastuzumab HC ORF (qPCR standard) | GAGGTTCAATTGGTTGAATCC | CTTACCAGGGGACAGAG |
| qPCR | trastuzumab LC ORF (qPCR standard) | GACATCCAAATGACTCAATCTC | ACACTCACCTCTGTTGAAG |

**Table S3**. GOseq gene set enrichment analysis for each phenotypic cluster under each condition.

**Condition A: 24h glycerol**

| Cluster 2 v. Cluster 1 | Y-17741 v. Cluster 1 | Y-11430 v. Cluster 1 | GS115 v. Cluster 1 | X-33 v. Cluster 1 |
|---|---|---|---|---|
| rRNA processing_1.00e-28 | | cytoplasmic translation_2.30e-32 | mitochondrial translation_1.40e-13 | |
| ribosomal large subunit biogenesis_2.16e-17 | | ribosome assembly_9.02e-06 | mitochondrion organization_5.55e-09 | |
| ribosomal small subunit biogenesis_4.29e-12 | | translational elongation_2.56e-04 | | |
| ribosome assembly_1.01e-05 | | | | |
| RNA modification_8.63e-05 | | | | |
| ribosomal subunit export from nucleus_1.71e-04 | | | | |

**Condition B: 48h glycerol**

| Cluster 2 v. Cluster 1 | Y-17741 v. Cluster 1 | Y-11430 v. Cluster 1 | GS115 v. Cluster 1 | X-33 v. Cluster 1 |
|---|---|---|---|---|
| | | | cellular amino acid metabolic process_0.00e+00 | |
| | | | tRNA aminoacylation for protein translation_3.08e-04 | |

**Condition C: 24h glycerol + 24h methanol**

| Cluster 2 v. Cluster 1 | Y-17741 v. Cluster 1 | Y-11430 v. Cluster 1 | GS115 v. Cluster 1 | X-33 v. Cluster 1 |
|---|---|---|---|---|
| cytoplasmic translation_1.63e-25 | cellular amino acid metabolic process_3.73e-04 | monocarboxylic acid metabolic process_9.37e-05 | cellular amino acid metabolic process_5.11e-21 | cellular respiration_1.49e-05 |
| cellular amino acid metabolic process_2.02e-07 | | | nucleobase-containing small molecule metabolic process_1.15e-05 | |
| ribosome assembly_3.69e-06 | | | tRNA aminoacylation for protein translation_2.30e-04 | |
| mut pathway_3.69e-05 | | | | |
| translational elongation_6.21e-05 | | | | |
| regulation of translation_2.89e-04 | | | | |
| vitamin metabolic process_3.20e-04 | | | | |

†Gene sets for which $p < 0.05$ are listed, following by the Bonferroni-corrected p-value.
‡Cluster 1 includes Y-48124, Y-12729, and Y-48123; Cluster 2 includes Y-7556/YB-4290, YB-378, and YB-4289. Differential expression analysis was performed relative to Cluster 1 in all cases.

**Table S4**. Top 20 loadings of Principal Component 2, as determined by principal component analysis of all strains under Condition A.

| PC2 loading | Gene ID | Gene Sym | PC1 loading |
|---|---|---|---|
| 0.084 | GQ67_05114 | - | -0.039 |
| 0.081 | GQ67_02654 | YDL218W | -0.013 |
| 0.067 | GQ67_04920 | - | 0.016 |
| 0.060 | GQ67_02261 | YFL040W | -0.026 |
| 0.052 | GQ67_00061 | NIT1 | -0.056 |
| 0.049 | GQ67_05174 | - | 0.004 |
| 0.047 | GQ67_00038 | - | -0.009 |
| 0.047 | GQ67_05243 | - | -0.010 |
| 0.044 | GQ67_04193 | - | -0.003 |
| 0.042 | GQ67_01392 | FLO11-BSC1 | -0.026 |
| -0.063 | GQ67_04750 | - | -0.021 |
| -0.064 | GQ67_03787 | - | -0.057 |
| -0.068 | GQ67_04316 | - | -0.037 |
| -0.070 | GQ67_02964 | - | -0.049 |
| -0.071 | GQ67_04497 | CTR1 | 0.001 |
| -0.078 | GQ67_04112 | FRE1 | 0.009 |
| -0.089 | GQ67_05224 | PDR12 | -0.017 |
| -0.090 | GQ67_02712 | - | -0.071 |
| -0.092 | GQ67_05326 | - | -0.060 |
| -0.113 | GQ67_03591 | ZPS1 | -0.070 |

**Table S5**. Reporter metabolite analysis on differentially expressed genes in GS115 relative to the other strains (X-33 and Y-48124 excluded).

**Condition B: 48h glycerol**

| NAME | Z-SCORE | P-VALUE | NEIGHBOURS | FUNCTIONAL GROUP |
|---|---|---|---|---|
| *2-Oxo-3-hydroxy-4-phosphobutanoate* | *7.21* | *2.81E-13* | *1* | *Pyridoxine Metabolism* |
| 3-Phosphohydroxypyruvate | 5.76 | 4.23E-09 | 2 | Glycine and Serine Metabolism |
| *O-Phospho-4-hydroxy-L-threonine* | *5.61* | *1.01E-08* | *2* | *Pyridoxine Metabolism* |
| 2-Oxoglutarate | 5.47 | 2.31E-08 | 25 | Transport, Nuclear |
| O-Phospho-L-serine | 4.94 | 3.84E-07 | 2 | Glycine and Serine Metabolism |
| *L-Glutamate* | *4.51* | *3.21E-06* | *55* | *Pyridoxine Metabolism* |
| Homocitrate | 3.85 | 5.99E-05 | 3 | Transport, Mitochondrial |

**Condition C: 24h glycerol + 24h methanol**

| NAME | Z-SCORE | P-VALUE | NEIGHBOURS | FUNCTIONAL GROUP |
|---|---|---|---|---|
| *2-Oxo-3-hydroxy-4-phosphobutanoate* | *6.30* | *1.52E-10* | *1* | *Pyridoxine Metabolism* |
| 3-Phosphohydroxypyruvate | 5.83 | 2.71E-09 | 2 | Glycine and Serine Metabolism |
| *O-Phospho-4-hydroxy-L-threonine* | *5.13* | *1.46E-07* | *2* | *Pyridoxine Metabolism* |
| Octadecynoyl-CoA | 4.68 | 1.45E-06 | 9 | Fatty Acid Biosynthesis |
| Tetradecenoyl-CoA | 4.68 | 1.45E-06 | 9 | Fatty Acid Biosynthesis |
| Hexadecenoyl-CoA | 4.68 | 1.45E-06 | 9 | Fatty Acid Biosynthesis |
| Octadecenoyl-CoA | 4.68 | 1.45E-06 | 9 | Fatty Acid Biosynthesis |
| *L-Glutamate* | *4.34* | *7.00E-06* | *55* | *Pyridoxine Metabolism* |

†Only metabolites with p-value < 1E-04 are listed; no metabolites met this criterion under Condition A.

**Table S6**. GOseq gene set enrichment analysis for transgenic strains.

### rhGH-expressing strains

| Y-48124 v. GS115 | Y-48124 v. Y-11430 | Y-48124 v. X-33 | GS115 v. Y-11430 | GS115 v. X-33 | Y-11430 v. X-33 |
|---|---|---|---|---|---|
| cellular amino acid metabolic process_1.59e-14 | | | cellular amino acid metabolic process_3.60e-11 | cellular amino acid metabolic process_6.06e-12 | |
| cytoplasmic translation_2.05e-09 | | | cytoplasmic translation_1.39e-05 | cytoplasmic translation_7.41e-09 | |
| ribosomal small subunit biogenesis_1.96e-04 | | | rRNA processing_3.69e-05 | ribosomal small subunit biogenesis_8.25e-06 | |
| | | | | rRNA processing_1.55e-05 | |

### rG-CSF-expressing strains

| Y-48124 v. GS115 | Y-48124 v. Y-11430 | Y-48124 v. X-33 | GS115 v. Y-11430 | GS115 v. X-33 | Y-11430 v. X-33 |
|---|---|---|---|---|---|
| cytoplasmic translation_2.56e-15 | methanol utilization pathway_2.47e-04 | | cellular amino acid metabolic process_3.2e-13 | cytoplasmic translation_4.59e-14 | |
| cellular amino acid metabolic process_1.99e-11 | | | | cellular amino acid metabolic process_5.03e-12 | |
| ribosomal small subunit biogenesis_1.25e-07 | | | | | |
| rRNA processing_1.3e-07 | | | | | |
| ribosomal large subunit biogenesis_7.95e-05 | | | | | |

†Gene sets for which $p < 0.05$ are listed, following by the Bonferroni-corrected p-value.
‡Clones of a given strain were grouped together for differential expression analysis.
